# Single- Versus Dual-Access Transcatheter Aortic Valve Implantation Using Balloon-Expandable Platform: A Propensity Score Matching Study

**DOI:** 10.1016/j.jacadv.2025.102086

**Published:** 2025-08-21

**Authors:** Muntaser Omari, Mario E. Diaz Nuila, Ahmed Abdalwahab, Debbie Stewart, Richard Edwards, Rajiv Das, Azfar Zaman, Mohamed Farag, Mohammad Alkhalil

**Affiliations:** a Cardiothoracic Centre, Freeman Hospital, Newcastle-upon-Tyne, United Kingdom; b Translational and Clinical Research Institute, Newcastle University, Newcastle-upon-Tyne, United Kingdom

**Keywords:** pacemaker, paravalvular regurgitation, single access, TAVI, VARC-3, vascular complication

## Abstract

**Background:**

Vascular complications are more frequently encountered in patients undergoing transcatheter aortic valve implantation (TAVI) compared to surgical aortic valve replacement. Single access (SA) TAVI may reduce access-site complications, but this approach has not been widely studied.

**Objectives:**

The aim of the study was to compare clinical outcomes of SA vs dual access (DA) TAVI using balloon-expandable valve Sapien 3 Ultra.

**Methods:**

This was a retrospective analysis of consecutive patients who underwent TAVI using balloon-expandable valve. Data were prospectively entered into a dedicated web-based database. The co-primary endpoints were device success and 30-day safety endpoints, defined according to the Valve Academic Research Consortium 3. A propensity score matching with a 1:1 ratio was performed comparing SA vs DA TAVI.

**Results:**

Of 532 patients who had transfemoral TAVI, 117 (22%) underwent the SA approach. Propensity score matching analysis yielded a well-balanced 204 patients (102 in each group). SA was associated with a significant reduction in contrast use, fluoroscopy time, radiation exposure, and total procedure time. Device success was comparable between the 2 groups (3.9% vs 3.9%; OR: 1.00; 95% CI: 0.24-4.11; *P* > 0.99). The 30-day safety profile of the Valve Academic Research Consortium 3 was significantly better in patients who underwent SA vs DA TAVI (15.0% vs 28.9%; OR: 0.44; 95% CI: 0.22-0.88; *P* = 0.02). This was derived from a lower incidence of severe paravalvular regurgitation and new pacemaker implantation.

**Conclusions:**

SA is a feasible and safe strategy compared to DA TAVI. It was associated with quicker procedure times, lower contrast and radiation exposure, and a better 30-day safety profile than the DA strategy. Future randomized studies are needed to further assess this strategy.

Transcatheter aortic valve implantation (TAVI) is increasingly considered the standard treatment for patients with severe aortic stenosis who are at intermediate or high surgical risk.^1^ Refinement of the transcatheter heart valve delivery system improved the safety profile and procedural success of TAVI and allowed direct comparison with surgical aortic valve replacement (SAVR), with TAVI demonstrating superiority over SAVR in recent trials at 1-year follow-up.^2^, ^3^, ^4^, ^5^

Major vascular complications, however, are more frequently encountered in patients undergoing TAVI compared to SAVR.^4^^,^^5^ The recent DEDICATE trial highlighted more than a 10-fold increase in incidence of vascular access-site complications with TAVI compared to SAVR (HR: 10.64; 95% CI: 4.84-28.94).^4^ Over the years, different strategies have been developed to reduce the risk of vascular complications during TAVI.^6^, ^7^, ^8^ Importantly, use of second vascular access is recognized as a significant contributor to access-site vascular complications, particularly when used via the contralateral femoral artery.^9^^,^^10^

Eliminating the second vascular access has the potential to reduce access-site complications but may compromise the ability to align the transcatheter heart valve during deployment. A previous study highlighted the feasibility of using single vascular access to perform TAVI, but it was a small study that did not allow for direct comparison between the 2 approaches (single access [SA] vs dual access [DA]) after adjustment for differences in baseline characteristics.^11^

The aims of this study were to determine the safety of using SA vs DA TAVI in patients undergoing balloon-expandable valve (BEV) and to evaluate in-hospital complications, beyond bleeding and access-site vascular complications, between the 2 strategies.

## Methods

### Study design

This is a retrospective analysis of consecutive patients who underwent TAVI procedure with a BEV using Sapien Ultra. All patients provided written informed consent for their procedures. Data were prospectively entered into a dedicated web-based database that was reviewed and maintained by a data manager who overlooked the database to ensure data accuracy and completion. Anonymized clinical, procedural, and echocardiographic data were collected from this database for analysis. The study was part of a clinical audit, and, therefore, it was exempted from the institutional review board, and the formal ethics process was waived. The study was conducted according to the Declaration of Helsinki and Good Clinical Practice guidelines.

### Study population

Between January 2022 and August 2024, consecutive patients with severe native aortic stenosis or a degenerative surgical bioprosthesis valve who were scheduled for TAVI procedure using BEV with Sapien 3 Ultra were included. All patients were discussed at and approved for TAVI by the local multidisciplinary team meeting in accordance with current guidelines.^1^ Patients with true low-flow aortic stenosis and an aortic valve calcium score of more than 1,200 Agatston units for women or 2,000 Agatston units for men were also included in this study. All patients underwent multidetector computed tomography as part of their procedural planning, and images were analyzed using 3mensio software (3mensio Structural Heart, 3mensio Medical Imaging), as previously described.^12^^,^^13^

### TAVI procedure

All patients underwent a percutaneous transfemoral approach under local anesthesia and conscious sedation by 2 experienced operators. The decision to undergo SA vs DA was left to the operator's discretion. From January 2022, some operators moved to routine (ie, no preselection) SA, while other operators continued with DA, performing SA in a selected group of patients according to their discretion.

**Figure 1:**
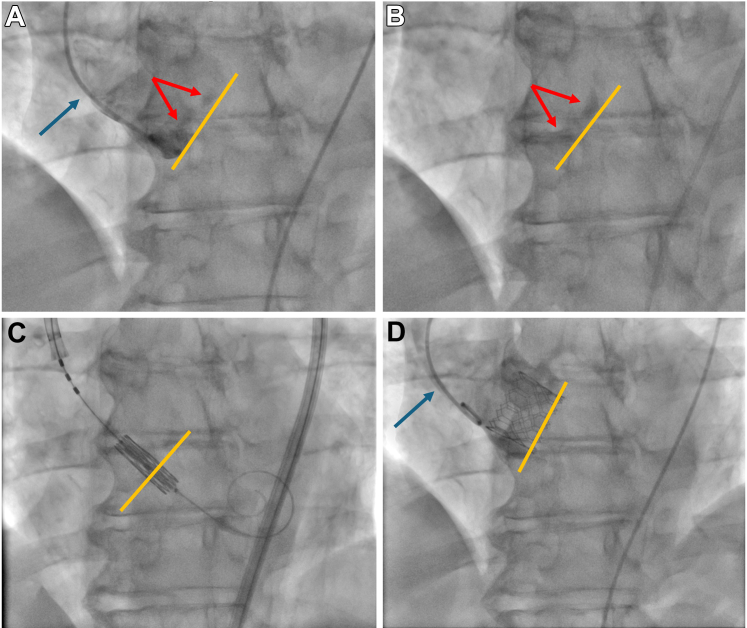
**Procedural Steps for Single Access Transcatheter Aortic Valve Implantation** Panel A demonstrates the position of the pigtail catheter in the noncoronary cusp (blue arrow) and aortogram highlighting the annular plane (yellow line) as well as aortic valve leaflet calcifications (red arrows). Panel B reflects an image acquisition without the pigtail with visible landmarks of both the annular plane and aortic valve leaflet calcifications. Panel C illustrates the positioning of the transcatheter heart valve using the calcified landmarks for valve placement. Panel D shows the final aortogram demonstrating high positioning of the transcatheter heart valve.

The DA access TAVI procedure was performed in line with the benchmarking standards for TAVI in the UK, as previously described.^12^^,^^13^ The SA approach involved inserting 14/16F Edwards E-Sheath after deploying Perclose ProGlide (Abbott Laboratories) closure devices. An aortogram was performed using a 5/6F pigtail catheter inserted through the Edwards E-Sheath and positioned in the noncoronary cusp. The aortic valve was subsequently crossed after removing the pigtail, and a dedicated TAVI wire was placed within the left ventricle (LV). The acquired aortogram was used as a roadmap to place the transcatheter heart valve and, where visible, using the aortic valve calcifications as a landmark for optimal positioning (Figure 1). In the absence of visible aortic valve calcification, a parallel wire technique was used by inserting a second wire inside the Edwards E-Sheath and leaving it in the noncoronary cusp as a landmark, as previously described.^14^ The transcatheter heart valve was deployed under rapid pacing using the LV wire. A final aortogram was performed after removing the valve delivery system to assess paravalvular regurgitation. If needed, postdilation was performed after recrossing the transcatheter heart valve. Both predilation and postdilation were performed according to the operator's discretion.

### Study endpoints

The primary endpoint was technical success at the exit from the procedural room according to the Valve Academic Research Consortium 3 (VARC-3).^15^ This was defined as freedom from mortality and surgical intervention related to the transcatheter heart valve, major vascular, access site, or cardiac complications. The other co-primary endpoint was VARC-3 early safety at 30 days, defined as freedom from all-cause mortality, all stroke, VARC type 2-4 bleeding, major vascular, access-related, or cardiac structural complication, acute kidney injury stage 3 or 4, severe aortic regurgitation, or new permanent pacemaker. Paravalvular regurgitation was categorized using the 3-class grading system (mild, moderate, and severe), and severe paravalvular regurgitation was defined using Doppler parameters of the VARC-3 criteria.^15^ The secondary endpoints included any vascular complications, any bleeding events, procedural duration, radiation exposure, and individual components of the co-primary endpoints.

### Statistical analysis

Data were tested for normality using the Shapiro-Wilk test and the unpaired *t*-test was used for comparison of normally distributed (presented as mean ± SD), and the Wilcoxon rank sum test was used for non-normally distributed variables (presented as median [IQR]). Categorical variables were compared using chi-square test or Fisher exact test, as appropriate. A propensity score matching with 1:1 ratio was performed to assess the role of SA approach in device success and 30-day safety endpoints using VARC-3 criteria. The propensity scores were estimated using a multivariable logistic regression model of SA vs DA TAVI. The following variables were used based on the best clinical judgment and relevance, including age, gender, body mass index, history of percutaneous coronary intervention, previous stroke or transient ischemic attack, peripheral vascular disease, frailty, valve size, and LV function with match tolerance of 0.02 (Supplemental Figure 1). A *P* value of <0.05 was considered significant. All statistical analyses were performed using SPSS 29.0 (SPSS, Inc).

## Results

Over the study period, 532 patients with severe aortic stenosis underwent transfemoral TAVI under local anesthesia using BEV with Edwards Sapien 3 Ultra. Of those, 117 (22%) patients underwent their procedure using the SA approach. There were no cases of crossover from the SA to the DA group. The mean age was 81 ± 7 years with 64% male. The mean gradient was 46 ± 15 in the whole cohort, and 71% had preserved LV function (ejection fraction of more than 50%). There was no difference in age and baseline clinical and echocardiographic characteristics between SA vs DA TAVI groups, including history of peripheral vascular disease, frailty, bicuspid valve morphology, or valve-in-valve procedure (Table 1). However, patients in the SA group were more likely to be male (82% vs 59%, *P* < 0.001). The propensity score match analysis yielded 204 well-matched patients, of which 102 underwent SA TAVI (Table 1).

**Table 1:** Baseline Clinical and Echocardiographic Characteristics of Included Patients

	Whole Cohort (N = 532)	Unmatched Cohort			Matched Cohort		
		Dual Access (n = 415)	Single Access (n = 117)	*P* Value	Dual Access (n = 102)	Single Access (n = 102)	*P* Value
Age, y (mean ± SD)	81 ± 7	81 ± 7	81 ± 8	0.40	79 ± 8	81 ± 8	0.27
Male, n (%)	340 (64%)	244 (59%)	96 (82%)	<0.001	86 (84%)	83 (81%)	0.58
Body mass index, kg/m^2^ (mean ± SD)	28 ± 6	28 ± 6	27 ± 6	0.29	27 ± 5	27 ± 6	0.55
Elective admission, n (%)	382 (72%)	302 (73%)	80 (68%)	0.35	73 (72%)	73 (72%)	>0.99
Diabetes, n (%)	149 (28%)	118 (28%)	31 (27%)	0.68	34 (33%)	25 (25%)	0.17
Hypertension, n (%)	328 (62%)	253 (61%)	75 (64%)	0.54	63 (62%)	61 (60%)	0.77
Previous smoker, n (%)	204 (38%)	159 (38%)	45 (39%)	0.98	52 (51%)	39 (38%)	0.067
Obstructive lung disease, n (%)	99 (19%)	81 (20%)	18 (15%)	0.31	20 (20%)	15 (15%)	0.35
Severe liver disease, n (%)	13 (2%)	12 (3%)	1 (1%)	0.21	4 (4%)	1 (1%)	0.17
Creatinine (mean ± SD)	110 ± 71	108 ± 67	116 ± 81	0.26	123 ± 108	118 ± 85	0.72
Hemoglobin (mean ± SD)	127 ± 20	129 ± 22	127 ± 19	0.41	126 ± 20	129 ± 22	0.31
Coronary artery disease, n (%)	92 (28%)	78 (29%)	14 (25%)	0.55	24 (35%)	12 (24%)	0.19
Previous PCI, n (%)	66 (12%)	57 (14%)	9 (9%)	0.09	12 (12%)	9 (9%)	0.49
Peripheral vascular disease, n (%)	67 (13%)	50 (12%)	17 (15%)	0.48	15 (15%)	15 (15%)	>0.99
Previous CVA/TIA, n (%)	67 (13%)	58 (14%)	9 (8%)	0.07	6 (6%)	7 (7%)	0.77
Previous CABG, n (%)	69 (13%)	57 (14%)	12 (10%)	0.32	19 (19%)	10 (10%)	0.071
Atrial fibrillation, n (%)	140 (26%)	26 (22%)	114 (28%)	0.26	34 (33%)	23 (23%)	0.086
NYHA functional class III/IV, n (%)	404 (76%)	314 (76%)	90 (77%)	0.78	80 (78%)	79 (78%)	0.87
Frail, n (%)	46 (9%)	32 (8%)	14 (12%)	0.15	10 (10%)	7 (7%)	0.45
Mean gradient (mean ± SD)	46 ± 15	46 ± 15	46 ± 14	0.57	43 ± 13	47 ± 13	0.074
Aortic valve area (mean ± SD)	0.71 ± 0.45	0.69 ± 0.18	0.80 ± 0.95	0.29	0.73 ± 0.17	0.81 ± 1.01	0.44
Bicuspid morphology, n (%)	12 (2%)	10 (2%)	2 (2%)	0.65	1 (1%)	2 (2%)	0.56
Valve-in-valve, n (%)	20 (4%)	17 (4%)	3 (3%)	0.44	5 (5%)	2 (2%)	0.25
Preserved LV function, n (%)	378 (71%)	293 (71%)	85 (73%)	0.67	63 (62%)	76 (75%)	0.051

CABG = coronary artery bypass graft; CVA = cerebrovascular event; LV = left ventricle; PCI = percutaneous coronary intervention; TIA = transient ischemic attack.

The right femoral artery was predominantly used in the whole cohort (85%) with no statistical difference between the SA and DA groups in both the matched and unmatched cohorts (Table 2). The number of patients who underwent predilation or postdilation was relatively small (<20%), with no differences between the SA and DA groups in the matched cohort. Similarly, valve size, predilation, and postdilation balloon sizes were comparable between groups in the matched cohort (Table 2). Contrast use (35 ± 21 mL vs 85 ± 43 mL, *P* < 0.001), fluoroscopy time (8.8 ± 5.4 minutes vs 10.7 ± 6.2 minutes, *P* = 0.036), radiation exposure (dose area product) (191 ± 148 Gy∗cm^2^ vs 253 ± 146 Gy∗cm^2^, *P* = 0.007), and procedural duration (54 ± 23 minutes vs 67 ± 27 minutes, *P* = 0.016) were statistically lower in patients who underwent SA vs DA TAVI. These findings were also seen in the unmatched cohort (Table 2).

**Table 2:** Procedural Characteristics of Included Patients

	Whole Cohort (N = 532)	Unmatched Cohort			Matched Cohort		
		Dual Access (n = 415)	Single Access (n = 117)	*P* Value	Dual Access (n = 102)	Single Access (n = 102)	*P* Value
Main access (right femoral), n (%)	443 (85%)	350 (86%)	93 (80%)	0.075	86 (86%)	82 (80%)	0.29
Secondary access (femoral), n (%)	-	58 (14%)	-	-	13 (13%)	-	
Predilation, n (%)	79 (15%)	63 (15%)	16 (14%)	0.69	16 (16%)	13 (13%)	0.55
Predilation balloon size, median (IQR)	18 (18-20)	18 (18-20)	20 (18-20)	0.13	20 (18-23)	20 (18-20)	0.48
Valve size, median (IQR)	26 (23-26)	26 (23-26)	26 (26-29)	<0.001	26 (26-29)	26 (26-29)	0.34
Postdilation, n (%)	72 (14%)	59 (14%)	13 (11%)	0.37	12 (12%)	12 (12%)	0.98
Postdilation balloon size, median (IQR)	26 (23-27)	24 (23-27)	26 (23-29)	0.27	27 (25-29)	26 (23-29)	0.36
Contrast use (mean ± SD)	74 ± 44	86 ± 41	37 ± 26	<0.001	85 ± 43	35 ± 21	<0.001
Fluoroscopy time (mean ± SD)	10.6 ± 5.9	11.1 ± 5.9	9.1 ± 5.5	0.003	10.7 ± 6.2	8.8 ± 5.4	0.036
Radiation dose (DAP) (mean ± SD)	235 ± 173	245 ± 175	199 ± 162	0.024	253 ± 146	191 ± 148	0.007
Procedural duration (mean ± SD)	62 ± 25	64 ± 26	53 ± 22	0.009	67 ± 27	54 ± 23	0.016

DAP = dose area product.

**Figure 2:**
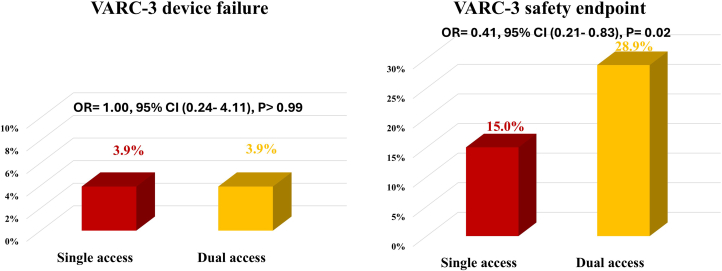
**The Incidence of the Primary Endpoints in the Study** The VARC-3 device failure and 30-day safety endpoint in patients who underwent single vs dual access TAVI. TAVI = transcatheter aortic valve implantation; VARC-3 = Valve Academic Research Consortium 3.

The first co-primary endpoint of device success at the exit from the procedure room was comparable between SA and DA strategies in both unmatched (4.3% vs 4.8%; OR: 0.88; 95% CI: 0.32-2.40; *P* = 0.81) and matched cohorts (3.9% vs 3.9%; OR: 1.00; 95% CI: 0.24-4.11; *P* > 0.99), respectively (Table 3, Figure 2). The second co-primary endpoint of VARC-3 30-day safety was lower in patients who underwent SA vs DA TAVI in both matched (15.0% vs 28.9%; OR: 0.44; 95% CI: 0.22-0.88; *P* = 0.02) and unmatched cohorts (16.2% vs 26%; OR: 0.55; 95% CI: 0.32-0.94; *P* = 0.03), respectively (Table 3, Figure 2).

**Table 3:** Clinical Outcomes of Included Patients, According to Vascular Access Strategy

	Whole Cohort (N = 532)	Unmatched Cohort			Matched Cohort		
		Dual Access (n = 415)	Single Access (n = 117)	*P* Value	Dual Access (n = 102)	Single Access (n = 102)	*P* Value
First co-primary endpoint^a^, n (%)	25 (4.7%)	20 (4.8%)	5 (4.3%)	0.81	4 (3.9%)	4 (3.9%)	>0.99
Second co-primary endpoint^b^, n (%)	127 (23.9%)	108 (26.0%)	19 (16.2%)	0.03	28 (28.9%)	15 (15.0%)	0.02
In-hospital death, n (%)	6 (1.1%)	5 (1.2%)	1 (0.9%)	0.75	1 (1%)	1 (1%)	>0.99
Major vascular complication, n (%)	11 (2.1%)	9 (2.2%)	2 (1.7%)	0.76	2 (2%)	1 (1%)	0.56
Any vascular complications, n (%)	26 (4.9%)	22 (5.3%)	4 (3.4%)	0.40	5 (4.9%)	2 (2%)	0.25
Neurological events, n (%)	23 (4.3%)	21 (5.1%)	2 (1.7%)	0.12	5 (4.9%)	2 (2%)	0.25
Stroke, n (%)	11 (2.1%)	10 (2.4%)	1 (0.9%)	0.30	2 (2%)	1 (1%)	0.56
Any bleeding events, n (%)	30 (5.6%)	25 (6%)	5 (4.3%)	0.47	5 (4.9%)	4 (3.9%)	0.73
Severe PVL, n (%)	13 (2.4%)	11 (2.7%)	2 (1.7%)	0.56	4 (4.1%)	0	0.04
New pacemaker, n (%)	35 (6.6%)	32 (7.7%)	3 (2.6%)	0.047	11 (10.8%)	3 (2.9%)	0.027
Mean gradient ≥20 mm Hg, n (%)	45 (10.3%)	37 (10.7%)	8 (8.9%)	0.62	8 (9.6%)	5 (6.3%)	0.44
30-d mortality, n (%)	10 (1.9%)	8 (1.9%)	2 (1.7%)	0.88	1 (1%)	2 (2%)	0.56

PVL = paravalvular leak.

a Defined technical success at the exit from the procedural room according to the Valve Academic Research Consortium 3 (VARC-3).

b This was defined as freedom from mortality and surgical intervention related to the transcatheter heart valve, major vascular, access site, or cardiac complications. The second co-primary endpoint was VARC-3 early safety at 30 days, defined as freedom from all-cause mortality, all stroke, VARC type 2 to 4 bleeding, major vascular, access-related, or cardiac structural complication, acute kidney injury stage 3 or 4, severe aortic regurgitation, or new permanent pacemaker.

There were no differences between groups in relation to in-hospital death, major or any vascular complications, bleeding or neurological events, or 30-day mortality. There were no cases of device embolization. However, SA was associated with a statistically significant reduction in new pacemaker implantation (2.9% vs 10.8%, *P* = 0.027) and incidence of severe paravalvular regurgitation (0% vs 4.1%, *P* = 0.04).

## Discussion

This study confirms the safety and feasibility of performing SA TAVI with BEV. The main findings from this study can be summarized as follows: First, SA was associated with reduced contrast load, radiation exposure, and procedure time; second, SA and DA TAVI were associated with comparable rates of device success according to the VARC-3 criteria; third, SA was associated with a better safety profile at 30 days; and finally, the difference in the VARC-3 30-day safety composite endpoints was derived from a significant reduction in new pacemaker implantation and lower incidence of moderate to severe paravalvular regurgitation (Central Illustration).

**Central Illustration:**
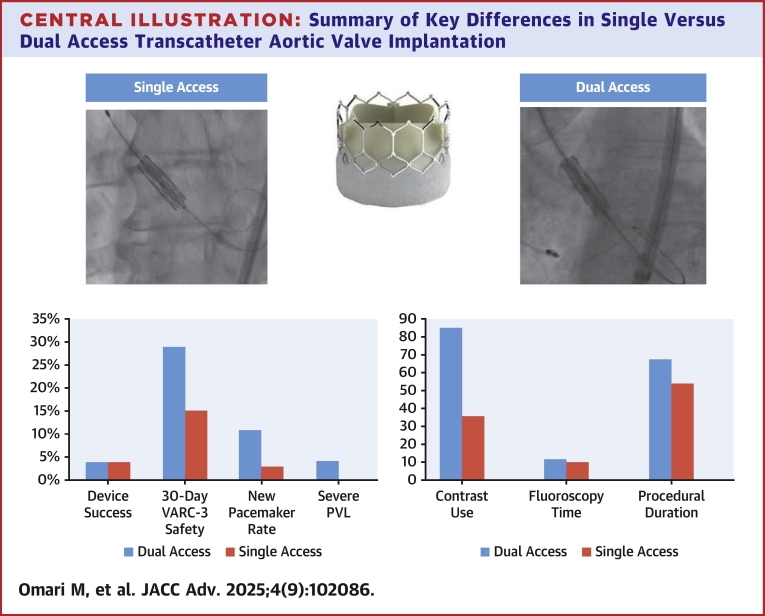
**Summary of Key Differences in Single Versus Dual Access Transcatheter Aortic Valve Implantation** The primary endpoint of device success was comparable between the 2 groups. The VARC-3 30-day safety profile was significantly better in patients who underwent SA vs DA TAVI, driven by lower pacemaker rate and paravalvular regurgitation. Contrast use, procedural duration, and fluoroscopy time were significantly lower in the SA group. DA = dual access; PVL = paravalvular leak; SA = single access; other abbreviations as in Figure 2.

Advances in transcatheter heart valve delivery systems have enabled a fully percutaneous procedure for TAVI. Numerous approaches were employed to minimize the risk of major vascular complications.^6^, ^7^, ^8^ These strategies focused on optimizing the antithrombotic status of patients pre- and post-TAVI procedures, including interrupting oral anticoagulation 48 hours preprocedure, routine administration of protamine at the end of TAVI, and a hybrid strategy of closure devices. Nonetheless, the use of a second vascular access, particularly the contralateral femoral artery, accounted for 25% of all vascular complications associated with TAVI.^11^ Therefore, minimally invasive TAVI emerged as a possible strategy to reduce access-site vascular complications. This strategy involves performing all procedural steps via single arterial access and eliminating the risks associated with the use of second vascular access during valve deployment.^11^^,^^16^^,^^17^

A study by Aroney et al^11^ highlighted the safety profile of using SA TAVI with a comparable rate of major vascular complications and a numerically lower incidence of minor vascular complications in patients undergoing SA TAVI. These findings were underscored in the retrospective multicenter registry PULSE registry (Plug or sUture based vascuLar cloSurE after TAVI).^18^ Our study highlighted similar findings of comparable rates of both major or any vascular complications. It demonstrated the safety profile of an SA approach by reporting a similar rate of device success when compared to DA TAVI. Moreover, the 30-day composite safety endpoint, defined according to the VARC-3 criteria, was significantly lower in the SA compared to the DA strategy. This observation was true in both the unmatched and matched propensity score cohorts. Nonetheless, the observational nature of our study would not allow us to ascertain causality. We can only speculate that SA may lead to better 30-day safety outcomes in patients undergoing TAVI with BEV Sapien 3 Ultra. Importantly, the use of the SA approach was irrespective of the degree of aortic valve calcifications on fluoroscopy, and the operators decided to proceed based on multidetector computed tomography images. The advent of the parallel wire technique has enabled the use of SA in patients with minimal aortic valve calcifications and extended its applicability to all patients who are scheduled for the TAVI procedure.^14^

Notably, valve size was statistically different between the 2 groups in the unmatched cohort. Patients in the DA group had smaller valve sizes compared to those in the SA one. The size of the valve was determined using the sizing chart, which makes this observation more likely to be related to a chance. More importantly, the difference in valve size was no longer present in the matched cohort, which may support our assumption.

Our data recorded lower rates of new pacemakers and paravalvular regurgitation. Aroney et al^11^ reported a lower incidence of mild paravalvular regurgitation with SA compared to DA TAVI. There was almost a 60% to 70% reduction in the incidence of pacemaker and paravalvular regurgitation in our study. This was evident, even after adjustments for the imbalance between the 2 approaches in a propensity score match analysis. We hypothesized that such a difference might be related to the final positioning of the transcatheter heart valve during deployment. Using the SA approach, it is likely that placement of the balloon marker of the Edwards Sapien 3 Ultra is adjusted based on the calcifications of the aortic valve leaflets rather than the true annulus. This might have resulted in higher implants and subsequently a lower incidence of new pacemaker implants. It is recognized that higher implantation of transcatheter heart valve relative to the aortic annulus is significantly associated with a reduced risk for pacemaker implantation.^19^ Moreover, high implants would enable the entire length of the outer skirt to interact with any regurgitant blood flow between the prosthesis and the aortic annulus, thus maximizing the opportunity to reduce paravalvular regurgitation.^20^ Nonetheless, these findings should be considered hypothesis-generating, and definitive conclusions cannot be established. Additionally, we did not correct for multiple comparisons, which can increase the risk of type 1 error. An appropriately sized randomized trial comparing SA vs DA is required to confirm these findings.

The elimination of secondary vascular access would restrict the operator from performing additional aortogram, and therefore the use of contrast was significantly lower in patients who underwent SA TAVI. While this may be protective against acute kidney injury, the overall use of contrast was relatively modest in both groups. Other mechanisms, beyond significant contrast use, such as hemodynamic instability during valve deployment or secondary to bleeding or major vascular complications, are more likely to lead to renal impairment following TAVI procedure.^21^ Additionally, the strategy of SA was associated with shorter fluoroscopy and procedure times as well as radiation exposure. A second vascular access would inevitably add to the procedure time and significantly increase radiation exposure and fluoroscopy time, particularly when using the radial artery. In the DA group, the radial artery was predominately used, and navigating the tortuosity of radio-brachial and subclavian arteries in an elderly population would increase radiation and procedure time. More importantly, it may explain the lack of a significant difference in vascular or bleeding complications, as radial is inherently safer than femoral access. Our data suggests that the use of the large-bore E-Sheath to insert the pigtail catheter, perform an aortogram, and plan the TAVI procedure using aortic landmarks was favorable to both the patient (less contrast and procedure time) and the operator (less radiation exposure) without compromising valve position at implantation.

### Study Limitations

Our study has several limitations that need to be highlighted. First, this is a single-center study with longstanding practice of using the SA approach. Therefore, results may not be generalized to centers with different procedural expertise. Second, our data were not randomized, and causal inferences cannot be applied to our results. Third, the decision to proceed with SA vs DA was determined by operator discretion and could have introduced unmeasured confounders. We have attempted to minimize any potential bias by conducting propensity score matching analysis to account for any imbalance between groups, but this would not eliminate selection or operator bias. Fourth, there was a lack of power calculation, which made it difficult to ascertain the relevance of nonsignificant findings. Finally, our findings are only applicable to the use of BEV with Sapien 3 Ultra. Other platforms using self-expanding valves require second vascular access for optimal valve positioning.

## Conclusions

The SA approach for TAVI is a feasible and safe strategy with similar device success compared to DA TAVI. It was associated with a reduction in contrast use, fluoroscopy and procedural times, and radiation exposure and demonstrated better 30-day safety profile than DA strategy. While we did not see a difference in vascular access complications, the reduction in overall procedure time coupled with reduced contrast and radiation exposure has potential benefits for health care providers, patients, and operators, respectively.

Perspectives **COMPETENCY IN MEDICAL KNOWLEDGE:** This is the largest study comparing clinical outcomes of SA vs DA TAVI. Device success was similar between the 2 groups, while the VARC-3 30-day safety profile was significantly better in patients who underwent SA TAVI. Likewise, there was a significant reduction in contrast use, fluoroscopy time, radiation exposure, and total procedure time associated with the use of SA TAVI.

**COMPETENCY IN PATIENT CARE AND PROCEDURAL SKILLS:** SA TAVI is safe and is associated with better clinical outcomes compared to DA TAVI. A high degree of procedural expertise is required prior to applying this technique to all-comers.

**TRANSLATIONAL OUTLOOK:** Future randomized studies are needed to confirm the above findings.

## Funding support and author disclosures

The authors have reported that they have no relationships relevant to the contents of this paper to disclose.
