# Supplementary material for: Single- Versus Dual-Access Transcatheter Aortic Valve Implantation Using Balloon-Expandable Platform: A Propensity Score Matching Study
Source: JACC Adv. 2025 Aug 21;4(9):102086. doi: 10.1016/j.jacadv.2025.102086 (PMC12398850; doi:10.1016/j.jacadv.2025.102086)

**Supplemental Figure 1: Boxplot of propensity score in both matched and unmatched cohorts of patients with single versus dual access TAVI.**


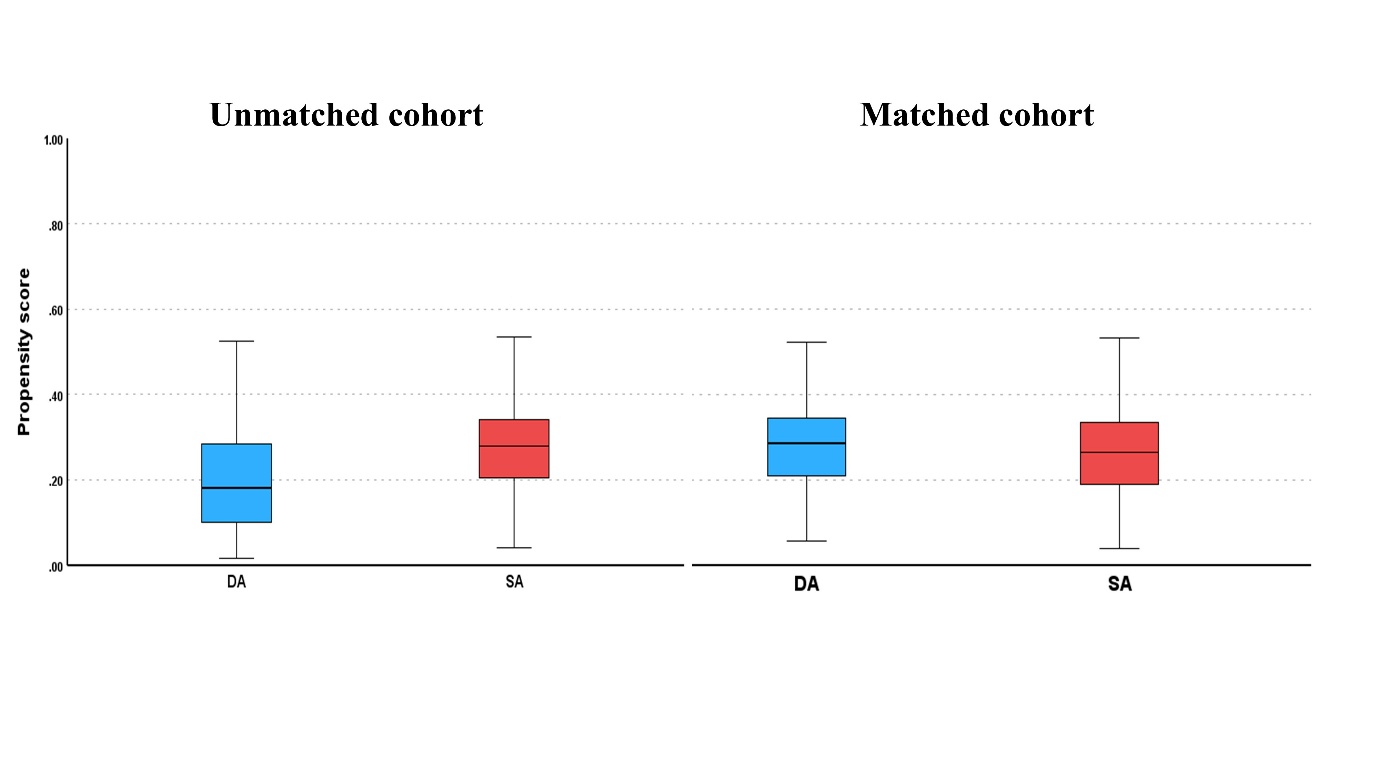

Supplement: Supplementary data [file mmc1.docx]
